# Lipidome and Gene Expression Profiling in Zebrafish Liver Spheroids: A 3D Model for Environmental Toxicology Applications

**DOI:** 10.1021/acs.est.5c02300

**Published:** 2025-06-13

**Authors:** Tiantian Wang, Miquel Perelló Amorós, Cinta Porte

**Affiliations:** † Environmental Chemistry Department, IDAEA − CSIC, C/Jordi Girona, 18-26, Barcelona 08034, Spain; ‡ PhD Program Aquaculture, University of Barcelona, Av.Diagonal 643, Barcelona 08028, Spain

**Keywords:** 3D cell culture, membrane lipids, polyunsaturated fatty acids, fatty acid synthesis, gene expression

## Abstract

Three-dimensional (3D) cell cultures offer more physiologically relevant models than conventional cell monolayers (2D) for mimicking in vivo tissue architecture. Despite their advantages, the use of 3D fish cell models in toxicological studies remains limited. This study aims to characterize the lipidomic and gene expression profiles of zebrafish liver (ZFL) spheroids and evaluate their metabolic and functional differences compared to those of conventional monolayer cultures. Seven-day-old spheroids (400 μm diameter) exhibited increased levels of neutral lipids—cholesterol esters (CEs) and diacylglycerols (DGs)—alongside reduced expression of genes involved in fatty acid synthesis (*fasn*, *acc*, and *scebf1*) compared to cell monolayers. This suggests a metabolic shift from lipid synthesis to lipid storage. Additionally, spheroids showed a reorganization of membrane lipids, with an increased ratio of phosphatidylcholines (PCs) to phosphatidylethanolamines (PEs) and elevated levels of phospholipids containing long-chain polyunsaturated fatty acids such as docosahexaenoic acid (DHA, 22:6). Upon exposure to β-naphthoflavone (BNF), the spheroids showed a markedly higher expression of *cyp1a* (650-fold) compared to monolayers (57-fold increase), indicating increased metabolic competence. Overall, ZFL spheroids represent a more mature and functionally relevant hepatocyte model than cell monolayers, particularly in terms of lipid composition and metabolic function. The study highlights the importance of lipidomic studies in characterizing 3D models and provides valuable insights into molecular changes associated with spheroid maturation.

## Graphical abstract

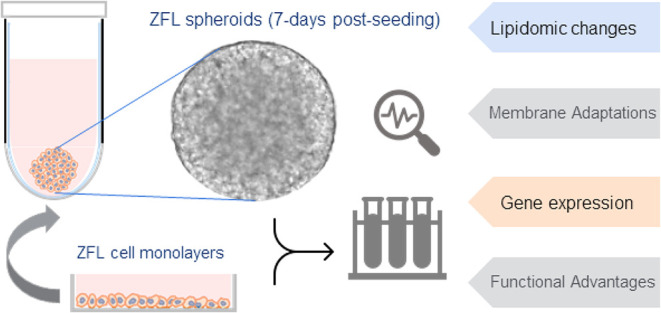

## 1 Introduction

In recent decades, breakthrough advances in biomedical research have transformed drug discovery and are slowly transforming toxicology approaches. The understanding of cellular mechanisms, such as signaling pathways and molecular interactions, has greatly improved risk and safety assessments of chemicals and pharmaceuticals.^1,2^ While animal testing remains valuable, its high costs and ethical concerns reinforce the need for alternative methods.^3,4^ Traditional 2D cell cultures, though widely used, are limited by their inability to mimic complex cellular environments, their reduced metabolic efficiency, and their shortened lifespan. As a result, 3D cell cultures have emerged as promising alternatives to more closely replicate in vivo conditions, offering a better reflection of cellular responses to external stressors and toxicants.^5^ Given the liver’s central role in detoxification and the elimination of harmful substances, various liver cell types, including primary hepatocytes, cancer-derived cell lines, immortalized hepatic cells, and stem cell-derived hepatocyte-like cells, have been cultured in 3D systems.^6^

3D models, including spheroids, organoids, and perfusion-based systems, enable interactions between cells and the extracellular matrix and support hepatocyte maturation.^7^ These models are often shown to mimic normal liver function and metabolic activity better compared to cell monolayers. For example, HepG2 spheroids exhibit enhanced drug-metabolizing capabilities, improved localization and function of nuclear receptors (CAR, PXR), and increased albumin secretion, leading to greater sensitivity for detecting toxicity.^6^ Similarly, HepaRG and HepG2 spheroids demonstrate enhanced expression of liver-specific genes, including several CYP450 isoenzymes.^8,9^ While the advantages of human cell spheroids are well-documented, the potential of 3D fish cell models remains largely unexplored, with only a handful of studies performed on freshly isolated hepatocytes or liver cell lines.^10–14^ The available studies show that fish liver cell spheroids exhibit comparable hepatic functions and metabolic capacities to those observed in vivo, making them valuable tools for evaluating the clearance of pharmaceuticals and chemicals.^13,15^ For instance, Baron et al.^16^ found that trout liver spheroids achieved intrinsic hepatic clearance rates for propranolol comparable to those reported in trout. More recently, scaffold-free 3D spheroid cultures derived from rainbow trout liver and kidney cell lines have been employed as a cost-effective method for cytotoxicity screening and for the propagation of fish viral pathogens.^14,17^

A deeper understanding of the molecular mechanisms underlying spheroid maturation and function can be achieved through lipidomic and transcriptomic profiling. These approaches provide valuable insights into key metabolic pathways, gene regulatory networks, and cellular adaptations that occur within 3D cultures, enhancing their functional relevance. Wang et al. (2023)^18^ demonstrated that PLHC-1 spheroids exhibit more mature lipid profiles than 2D cultures, characterized by an increased PC/PE ratio and elevated levels of highly unsaturated PCs and CEs, consistent with what is typically found in fish liver tissue. Additionally, the spheroids showed more pronounced changes in their lipidome compared to cells grown in monolayers when exposed to a mixture of plastic additives.^18^ Since lipid metabolism is crucial for maintaining energy balance, cell membrane integrity, and supporting cellular functions, understanding lipid signatures and their modulation by pollutant exposure is essential for accurately assessing the effects of chemicals.^19–21^ Thus, alterations in lipid composition and abundance often serve as valuable biomarkers of toxicity. For example, Pérez-Albaladejo et al.^22^ demonstrated that PLHC-1 cells (2D) exposed to phthalates, such as dibutyl phthalate (DBP) and di­(2-ethylhexyl) phthalate (DEHP), exhibited a significant accumulation of triglycerides (TGs), while chlorinated bisphenol A diglycidyl ether (BADGE·2HCl) led to a significant depletion of TGs, evidencing the different modes of action of plastic additives in topminnow liver cells. Additionally, Marqueño et al.^23^ showed the ability of bisphenol A derivatives to alter lipid homeostasis in zebrafish liver (ZFL) cells, with effects such as as dihydroceramide accumulation or the specific reduction of TGs containing highly unsaturated fatty acids as a consequence of oxidative stress.

Building on the relevance of 3D culture models in toxicology, this work aimed to investigate how culturing ZFL cells as spheroids influences their lipidome and metabolic activity compared to monolayer cultures and, consequently, their response to toxicants. To address this question, the lipidome of 7-day-old ZFL spheroids was analyzed with ultrahigh-performance liquid chromatography-high-resolution mass spectrometry (UHPLC-HRMS), with special attention paid to the fatty acid composition of membrane lipids, such as PCs and PEs. Additionally, we examined the expression of key genes involved in liver function and evaluated the metabolic response of ZFL spheroids to the cyp1A inducer β-naphthoflavone.

## 2 Material and Methods

### 2.1 Chemical and Solutions

Fetal bovine serum (FBS), HEPES, insulin, β-naphthoflavone (BNF, purity ≥ 98%), and dimethyl sulfoxide (DMSO) were obtained from Sigma-Aldrich (Merck, Darmstadt, Germany). Dulbecco’s Modified Eagle Medium:Nutrient Mixture F-12 (DMEM/F-12), Leibovitz L-15 medium, nonessential amino acids, penicillin, streptomycin, amphotericin B, and 0.25% trypsin-ethylenediaminetetraacetic acid (EDTA) were sourced from Gibco (Thermo Fisher Scientific, Inc., UK). Basic fibroblast growth factor (bFGF) was purchased from Thermo Fisher Scientific, Inc., UK. The LIVE/DEAD Viability/Cytotoxicity Kit was obtained from Invitrogen (Paisley, UK). Internal standards for lipidomic analyses, including 16:0-d31-18:1 PC, 17:0 lysophosphatidylcholine (LPC), C18 (Plasmalogen)-18:1 (d9) phosphatidylcholine (PC-P), 16:0-d31-18:1 PE, 16:0-d31-18:1 phosphatidylserine (PS), 16:0-d31-18:1 phosphatidylinositol (PI), d31-18:1 phosphatidylglycerol (PG), 16:0-d31 sphingomyelin (SM), 16:0-d31 ceramide (Cer), 1,3-17:0-d5 diacylglycerol (DG), 1,2,3-17:0 triacylglycerol (TG), and 16:0 cholesteryl-d7-ester (CE), were acquired from Avanti Polar Lipids, Inc. (Alabama, US).

### 2.2 Cell Culture Conditions and 3D Spheroid Formation

The zebrafish liver (ZFL) cell line, obtained from the American Type Culture Collection (ATCC CRL-2643), was cultured in 75 cm^2^ flasks (Corning, NY, USA) at 28 °C in a 5% CO_2_ humidified incubator. Cells derived from passages 5 to 25 were detached using 0.25% trypsin upon reaching confluence. ZFL cell monolayers, and spheroids were maintained in a complete growth medium (CGM) combining 50% DMEM/F12 with 50% Leibovitz-15 medium, supplemented with 5% FBS, 15 mM HEPES, 0.1 mM nonessential amino acids, 10 μg/mL insulin, 10 ng/mL bFGF, 100 U/mL penicillin, 100 μg/mL streptomycin, and 25 μg/mL amphotericin B, as described in previous protocols with some modifications.^15,24^

For the formation of ZFL spheroids, cells were plated in ultralow-attachment 96-well round-bottom plates (Corning, NY, USA) at 28 °C.^18^ Different cell densities (5,000 to 20,000 cells/well) were tested for spheroid size optimization. Cell clusters were formed overnight, and the growth of the spheroids was stimulated by changing half of the culture medium (CGM) every 2–3 days. All replicates were derived from ZFL cells between passages 10 and 25 to ensure consistency and reproducibility. Spheroid images were taken with an EVOS M7000 microscope imaging system (Thermo Fisher Scientific).

### 2.3 Spheroid Viability

The viability of spheroids was assessed through live/dead analysis as described by Wang et al. (2023),^18^ with some modifications. Spheroids were treated with 2 μM calcein-AM (CAM, green fluorescence) and 4 μM ethidium homodimer-1 (EthD-1, red fluorescence) for 40 min at 28 °C and 5% CO_2_. Subsequently, fluorescence microscopy was conducted using an EVOS M7000 Imaging System to visualize live (green) and dead cells (red). Z-stack images consisting of approximately 50 slices were captured, and these images were merged for the two fluorescence channels (GFP and RFP). The merged images were then used to analyze spheroid growth and changes in viability over time. Raw images were presented without any adjustments, and a scale bar was added using ImageJ software (v.1.53c).

### 2.4 Untargeted Lipidomic Analysis

ZFL cell monolayers were cultured in a 12-well plate (Nunclon Delta Surface, Thermo Fisher Scientific, Alcobendas, Spain) at a density of 1 × 10^6^ cells/mL/well in CGM for 48 h. Afterward, cells were rinsed with cold phosphate-buffered saline (PBS, 1×), harvested in PBS under ice-cold conditions using a rubber scraper, and centrifuged at 2,000 g for 15 min at 4 °C to obtain the cell pellets. The pellets were then stored at −80 °C under an argon atmosphere until further extraction. Similarly, ZFL spheroids (20,000 cells/well) were grown for 7 days in CGM, and 10 individual spheroids were pooled per sample. After being washed with PBS, the spheroid pellets were collected by centrifugation (2,000 g) and stored at −80 °C under an argon atmosphere until extraction. Three plates with two technical replicates per plate were analyzed (*n* = 6). All replicates were derived from ZFL cells between passages 10 and 25 to ensure consistency and reproducibility.

Cell pellets were thawed and extracted with ethyl acetate (3×) as described by Marqueño et al. (2019).^25^ Dried lipids were reconstituted in 300 μL (cell monolayers) and 100 μL (spheroids) of methanol for subsequent UHPLC-HRMS analysis, and lipid internal standards (50 to 200 pmol) were added (see Table S1 for details). Lipids were analyzed with an Elute UHPLC system coupled with an Impact II quadrupole time-of-flight (QToF) mass spectrometer (Bruker Daltonics, Bremen, Germany), as detailed by Hosseinzadeh et al.^26^ Initially, lipid extracts (10 μL) were injected into a 100 × 2.1 mm, 1.7 μm Acquity UPLC BEH C8 column (Waters, Ireland) at 30 °C. The mobile phase consisted of (A) water with 2 mM ammonium formate and 0.01% formic acid and (B) methanol with 1 mM ammonium formate and 0.01% formic acid. A constant flow rate of 0.25 mL/min was maintained, with a gradual transition in the organic gradient over 25 min: 0–0.6 min (80% B), 0.6–3 min (90% B), 3–6 min (90% B), 6–14 min (99% B), 14–19 min (99% B), 19–22 min (80% B), and 22–25 min (80% B). Untargeted mass acquisition, performed in positive electrospray mode, covered a mass-to-charge ratio (*m*/*z*) scan range of 200–1200, with mass spectra recorded at 12 Hz in auto MS/MS mode. Both external and internal mass calibrations relied on sodium formate ion clusters for accuracy and precision.

Lipidomic data were processed using MetaboScape (v. 2022, Bruker Daltonics) following Wang et al. (2023).^18^ Extraction of features was conducted using the T-Rex 3D workflow for peak picking and alignment. Feature detection was performed using an intensity threshold (>1000 counts), peak length (5 spectra), mass range (200 to 1200 *m*/*z*), and retention time (2–25 min). [M + H]^+^, [M + Na]^+^, and [M + NH_4_]^+^ ions were selected in the ion configuration settings. Annotation of lipids was performed using the algorithm MCube Lipid Species Annotation, which relied on accurate precursor ion mass, isotopic patterns (mSigma value), and MS/MS spectra according to lipid class-specific fragmentation rules. Lipid species were annotated using a standardized nomenclature indicating lipid class, total carbon number, and number of double bonds (e.g., PC 36:5), or specifying fatty acid composition when structural information was available (e.g., PC 16:0_20:5). Identified lipids were manually curated using the LipidMaps database and exported for further quantification. A total of 353 lipids, corresponding to 15 lipid families, were annotated and quantified, while 1200 features remained uncharacterized. The relative standard deviation (RSD) of internal standards was typically below 15%, except for DGs in spheroid lipid samples, which presented slightly higher values (25%). Lipid species were quantified based on their internal standards and corresponding adducts (Table S1), except for PE-plasmalogen/PE-plasmanyl (PE-P/PE-O), hexosylceramides (HexCer), and sphingoid bases (SPB), where PE, Cer, and SM standards were used, respectively. QA/QC processes included the use of a mixture of class-representative internal standards spiked prior to injection, allowing correction for ionization variability. Instrument performance was monitored through repeated analysis of a mixture of external standards injected at regular intervals, and consistency was assessed on the basis of their response and clustering in principal component analysis (PCA).

### 2.5 Real-Time PCR

Total RNA was extracted from six replicates of both cells grown in monolayers (12-well plate) and spheroids (7-day-old, 8 spheroids/sample) using Trizol, according to the manufacturer’s protocol (Ambion, Thermo Fisher Scientific). After washing with PBS, all samples were lysed in 1 mL of Trizol solution, transferred to microcentrifuge tubes, vortexed for one min, and stored at −80 °C overnight to enhance cell lysis and RNA release. Extraction proceeded the next day according to the manufacturer’s instructions. RNA purity was assessed using the NanoDrop 8000 UV–vis Spectrophotometer (Thermo Scientific). The 260/230 and 260/280 ratios ranged from 2.0 to 2.2 and 1.9 to 2.1, respectively, indicating good RNA purity with minimal protein or solvent contamination. The RNA concentration ranged between 188 and 679 ng/μL. Genomic DNA was removed with Ambion DNase I (Thermo Fisher Scientific), and RNA (1500 ng/sample) was reverse-transcribed to cDNA using the Transcriptor First Strand cDNA Synthesis Kit (Roche Diagnostics) according to the manufacturer’s protocol. Negative controls were prepared by replacing the RT enzyme with water. A set of 16 genes involved in liver function (*igf1*, *asl*, *cyp3a65*, *cyp1a*, *ugt1a1*, and *g6pd*), fatty acid biosynthesis (*fasn*, *acc*, *screbf1*, *elovl6*, and *scd*), and lipid storage/transport (*dgat1a*, *mtp*, *lact*, *scarb1*, and *abca1b*) were selected (see Table S2 for gene names and primer sequences). Quantitative real-time PCR was conducted using a LightCycler 480 Real-Time PCR System in a final volume of 10 μL using SYBR Green mix (Roche Applied Science, Mannheim, Germany), with primers added at a final concentration of 0.3 μM each. The housekeeping gene peptidylprolyl isomerase A (*ppiaa*) was used as the reference gene due to its minimal variability between samples from monolayers and spheroids and high amplification efficiencies (91.3% and 91.8%, respectively), supporting its suitability for normalization. The amplification protocol included an initial denaturation step at 95 °C for 10 min, followed by 45 cycles of amplification consisting of 10 s at 95 °C and 30 s at 60 °C. Melting curve analysis was performed by gradually increasing the temperature from 65 to 95 °C at a ramp rate of 0.11 °C per second. Once the temperature reached 95 °C, it was held for 2 s before cooling. Relative transcript abundance was calculated using the ΔΔCp method.^27^ The data are presented as fold change relative to the expression determined in cells grown in monolayers (*n* = 6).

To assess the inducibility of cyp1a expression, a hallmark response to xenobiotic activation of the aryl hydrocarbon receptor (AhR), spheroids and cell monolayers were exposed to 1 μM β-naphthoflavone (BNF), a well-characterized cyp1a inducer, or 0.1% DMSO (solvent control). The BNF concentration was selected based on previous literature showing effective cyp1a induction in PLHC-1 cells.^28^ Exposures were performed for 6 h in both monolayers and spheroids and extended to 24 h in spheroids only to evaluate the persistence of the response in the 3D model. Gene expression analysis was performed using three plates with two technical replicates each (*n* = 6). All replicates were derived from ZFL cells between passages 10 and 25.

### 2.6 Statistical Analysis

Statistical analyses were conducted using SPSS v27. The normality and homoscedasticity of the data were confirmed with the Shapiro-Wilk and Brown-Forsythe tests, respectively. Changes in spheroid size and differences in lipidomic profile and gene expression between cell monolayers and spheroids were evaluated using one-way ANOVA, followed by Tukey’s posthoc test (*p* < 0.05). Graphs were generated using GraphPad Prism 8.0.1 (GraphPad Software, San Diego, California, USA).

MetaboAnalyst 6.0 was utilized to analyze differences in the lipidome of ZFL cell monolayers and spheroids.^29^ Lipid features not detected in some samples due to signal intensities falling below the limit of detection were treated as missing values. These values were imputed by replacing them with one-fifth of the minimum positive value observed for each feature within the corresponding sample group. Lipid species with more than 50% missing values across all replicates were excluded from further analysis. The data were normalized by sum, autoscaled by centering around the mean, and divided by the standard deviation of each variable to approximate a normal distribution. Partial least-squares discriminant analysis (PLS-DA) was used to cluster samples based on lipidomic profiles. Model performance was assessed based on internal classification accuracy (*R*²) and predictive capability (*Q*²). Volcano plot analysis, combining fold change (FC > 2) and Student’s *t* test (*p*-value <0.05), was used to identify significant features from both biological and statistical perspectives.

BioPAN, an open-access tool available on the LIPID MAPS website (https://www.lipidmaps.org/biopan/), was used to identify active or suppressed lipid pathways in spheroids compared to cell monolayers, with a significance threshold of Z-score > 1.645 (equivalent to *p*-value < 0.05).^30^ Prior to analysis, lipid names were standardized to the LIPID MAPS nomenclature by using the LipidLynxX tool. Additionally, lipid results with fatty acid position and double bond location were converted into a sum composition of carbon number and double bond equivalents. For example, PC 16:0_20:5 and Cer 18:0;O2/16:0 were reformatted as PC(36:5) and dhCer(16:0), respectively.

## 3 Results

### 3.1 Spheroid Formation

Under the current culture conditions, ZFL cells seeded in ultralow attachment (ULA) plates at different densities (5,000–20,000 cells per well) initially formed loose cell aggregates, which progressively compacted into spheroids within 1 week, reaching diameters between 281 and 452 μm (Figure 1). Brightfield and live/dead fluorescence imaging at days 7, 14, and 21 postseeding revealed variations in spheroid size depending on the initial seeding density. Spheroids retained excellent cell viability and structural integrity at all time points and across all seeding densities, including the highest (20,000 cells per well). This was evidenced by the uniform and intense green fluorescence signal (live cells) and the near-complete absence of red fluorescence (dead cells), with no indication of necrotic core formation (Figure 1). To evaluate the reproducibility of spheroid formation, we standardized the seeding density at 20,000 cells per well and a culture period of 7 days. The reproducibility was confirmed by analyzing 10 spheroids per plate (*n* = 3), with low intraplate (1.4–2.7%) and interplate (2.9%) coefficients of variation, indicating high consistency and reliability of the spheroid generation technique.

**Figure 1:**
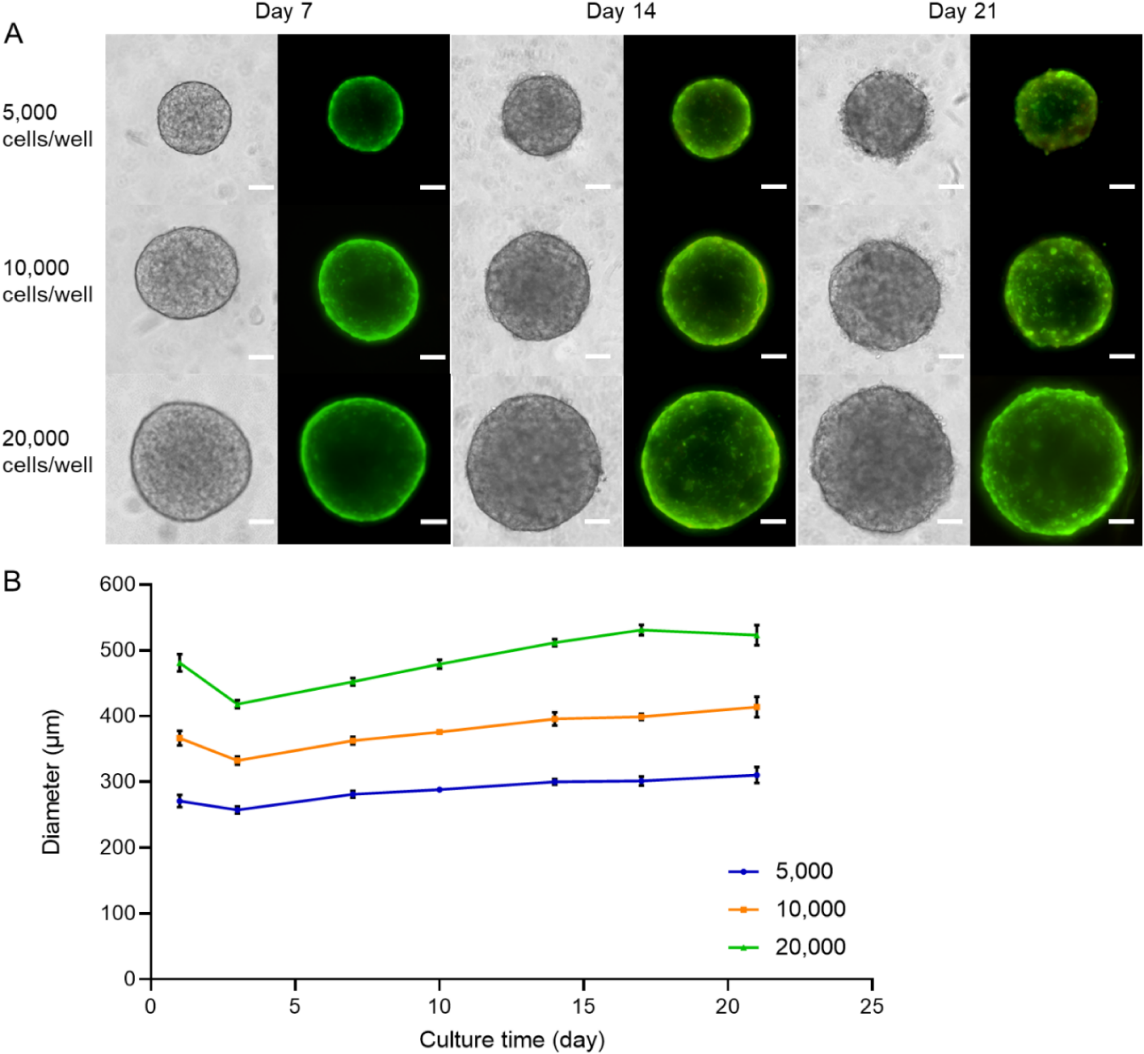
Growth and viability of ZFL spheroids as a function of initial seeding density (5,000 to 20,000 cells/well). (A) Representative images showing changes in morphology (brightfield images) and cell viability (live/dead staining) across multiple time points (days 7, 14, and 21 postseeding). Green fluorescence indicates live cells (calcein-AM), and red fluorescence indicates dead cells (ethidium homodimer-1). Scale bar: 100 μm. (B) Spheroid diameter measured over time. Values are the mean ± SD of 20 spheroids per condition.

### 3.2 The Lipidome of ZFL Spheroids vs Cell Monolayers

The lipidomic analysis revealed significant differences in the lipid profiles between ZFL cell spheroids and monolayers. In spheroids, there was a significant increase in CEs (from 1.0% to 14.9%), Cer (from 2.5% to 6.9%), HexCer (from 0.1% to 0.2%), DGs (from 5.7% to 7.7%), and LPCs (from 0.2% to 0.3%). Conversely, spheroids showed a significant reduction in certain membrane lipids, including PC-P/PC-Os (from 3.0% to 1.6%), PEs (from 21.6% to 13.1%), PE-P/PE-Os (from 23.0% to 10.4%), and SMs (from 3.9% to 2.8%) (Figure 2). Despite these changes, PCs remained the most abundant lipid species in both systems, accounting for 29.6% and 32.1% of the total identified lipids in cell monolayers and spheroids, respectively.

**Figure 2:**
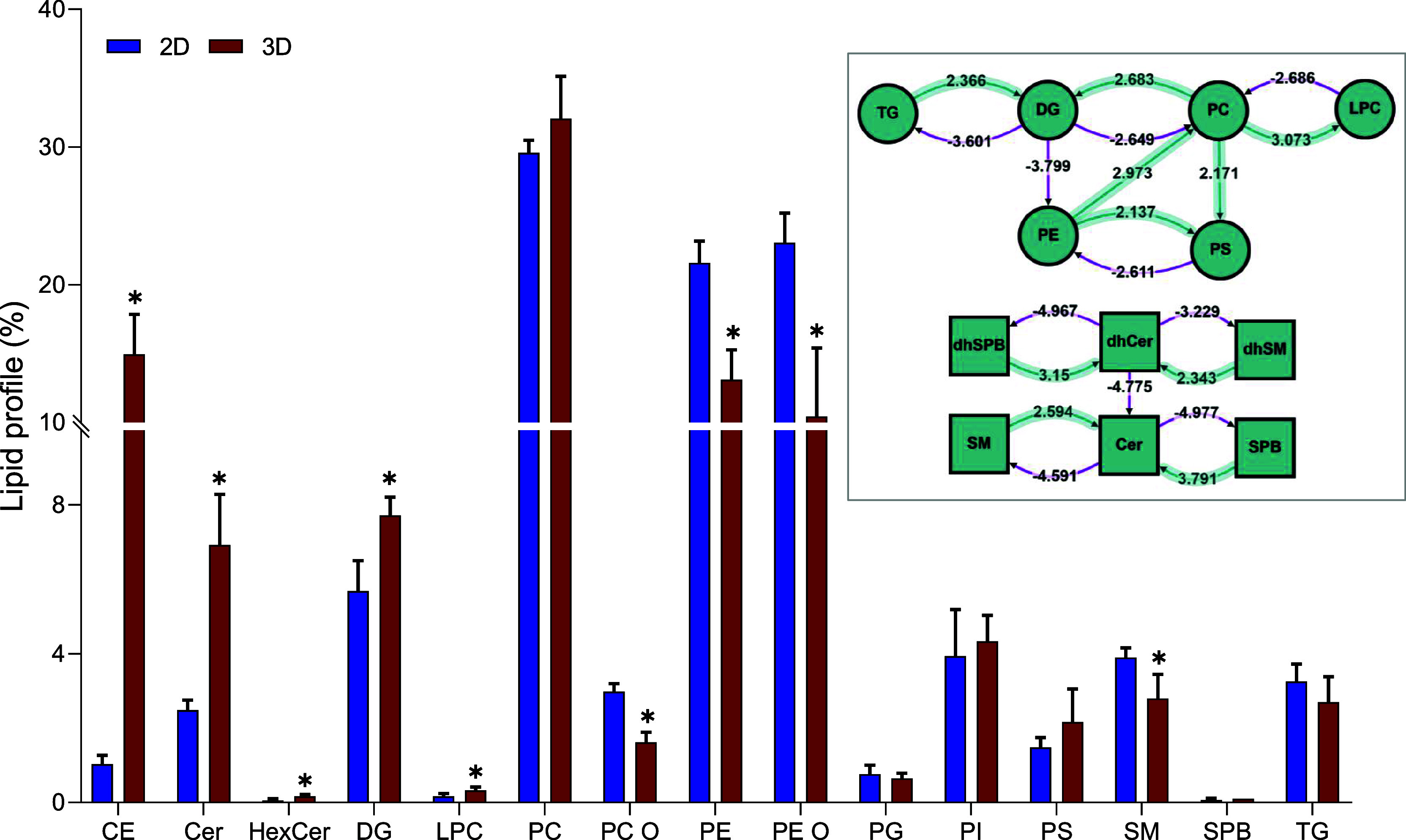
Lipid profile of ZFL cell monolayers (blue) and spheroids (red) presented as the percentage of each lipid subclass in the total identified lipids. Values are mean ± SD (*n* = 6). * denotes statistically significant differences (*p* < 0.05). BioPAN-activated pathways are indicated in the top right corner. Green arrows indicate the positive Z-score implied in an active status, purple arrows indicate the negative-Z-score.

PLS-DA analysis of all annotated lipids demonstrated a clear distinction between the lipidome of 7-day-old spheroids and ZFL cell monolayers, explaining 71.8% of the variance (*R* ^2^ = 0.96, *Q* ^2^ = 0.92) (Figure S1A). The variable importance in projection (VIP) scores highlighted the lipids driving this separation, with PC-Os/PC-Ps, CEs, DGs, SM, Cer, and PCs playing a key role in differentiating the two cell models (Figure S1A).

Volcano plots further highlighted statistically significant changes in individual lipids contributing to this separation. By setting a fold change greater than 2.0 and a *p*-value less than 0.05, 58 upregulated and 72 downregulated lipids were identified as significantly altered (Figure S1B). Several lipid subclasses exhibited particularly high fold changes, namely CEs (CE 18:2, 18:3, 20:4), Cer (Cer 18:0;O2/24:0, 44:1;O2), LPCs (LPC 16:0, 20:3), and ether-linked phospholipids (PC O-40:7, PC O-40:1; PE O-38:5, PE O-36:4) (Table S3).

BioPAN analysis provided insights into the metabolic pathways associated with the observed lipid changes. It revealed the activation of several lipid pathways in spheroids, including the hydrolysis of TGs → DGs; PEs → PCs → DGs; PEs → PCs → LPCs; PEs → PCs → PSs; PEs → PSs; SPBs → Cer; and SMs → Cer, leading to the intracellular accumulation of DGs, LPCs, PSs, and Cer (Figure 2). Among these, the hydrolysis of PEs → PCs → LPCs exhibited the highest Z-score (4.275), followed by PEs → PCs → DGs (3.99) and SPBs → Cer (3.79), making them the most significant lipid reactions activated in spheroids compared to cells grown in monolayers (Table S4). These active reactions suggest remodeling of phospholipid metabolism in spheroids, with increased activities of phospholipase A2 (PLA2), which cleaves phospholipids to generate LPCs and free fatty acids and likely contributes to the accumulation of LPCs, and increased phosphatidylethanolamine *N*-methyltransferase (PEMT), which catalyzes the methylation of PEs to produce PCs. Additionally, BioPAN analysis revealed the activation of several genes involved in sphingolipid metabolism, including CERS1-6, which encode ceramide synthases responsible for ceramide biosynthesis, and ASAH1, ASAH2, and ASAH2B, which encode acid and neutral ceramidases that hydrolyze ceramides into sphingosine and free fatty acids. These findings suggest a broader reorganization of lipid metabolism in spheroids, encompassing both phospholipid and sphingolipid pathways.

### 3.3 Changes in Membrane Lipid Composition in ZFL Spheroids

Culturing ZFL cells as spheroids, instead of monolayers, resulted in a significant increase in the PC/PE ratio, from 1.4 ± 0.1 to 2.5 ± 0.5. Additionally, the acyl chain composition and degree of unsaturation in both PCs and PEs underwent significant changes. Compared with cell monolayers, spheroids displayed an increase in both the fatty acid chain length and unsaturation level of PCs, while PEs primarily showed an increased abundance of species with very long-chain fatty acids (Figure 3). Among PCs, those with 32, 34, and 36 acyl carbons were predominant, comprising 82% and 80% of total PCs in 2D and 3D systems, respectively (Figure 3A). Notably, spheroids showed a reduction in saturated PCs (from 10.7 ± 2.1% in 2D to 4.7 ± 1.0% in 3D) (Figure 3B) and a marked increase in PCs containing long-chain PUFAs, such as arachidonic (20:4), eicosapentaenoic (20:5), and docosahexaenoic acid (22:6). Specifically, PC 16:0_20:5 increased 14.3-fold, PC 16:0_22:5 increased 10.5-fold, PC 18:2_20:4 increased 10.9-fold, and PC 18:0_22:6 increased 3.3-fold (Table S3). Some of these upregulated PCs, such as PC 16:0_20:5, were scarcely present in 2D cells (Figure 4A).

**Figure 3:**
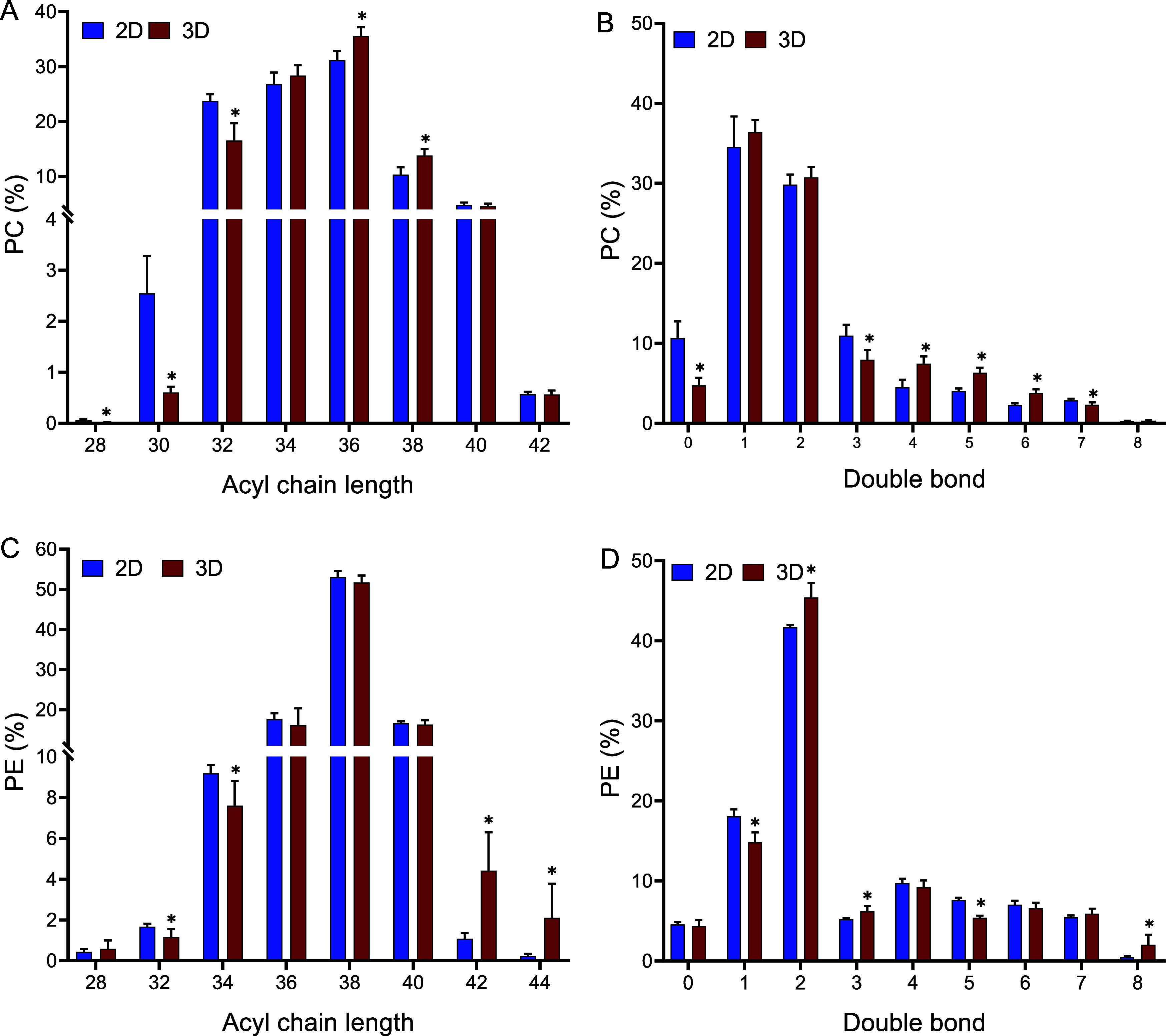
Changes in PC and PE acyl chain length (A, C) and degree of unsaturation (B, D) in ZFL cell monolayers and spheroids. Values are mean ± SD (*n* = 6). *Indicates statistically significant differences (*p* < 0.05).

**Figure 4:**
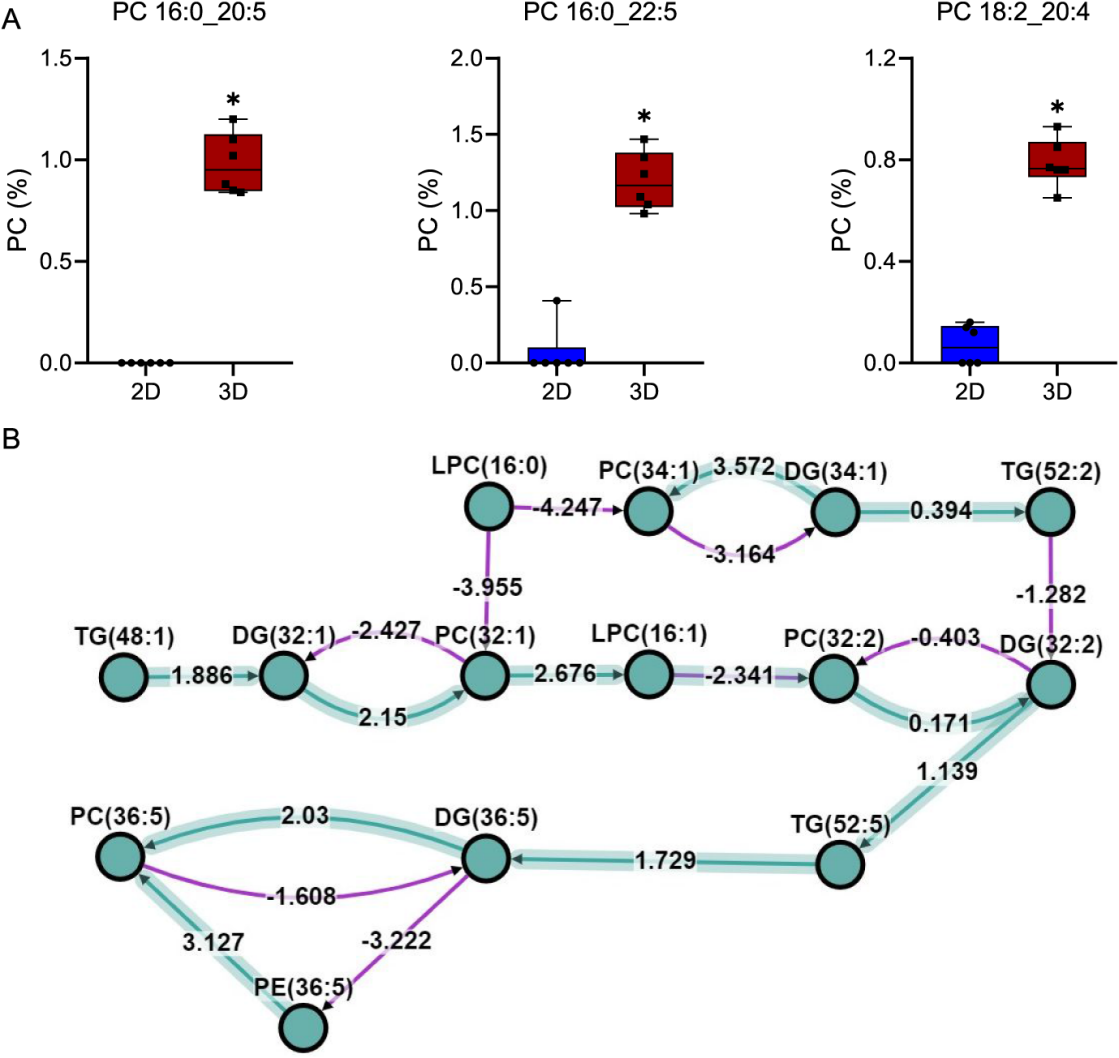
Key changes in highly unsaturated PCs of ZFL spheroids compared to cell monolayers (A), and representative BioPAN-activated reactions (B). Values indicate the percentage of total PCs, presented as mean ± SD (*n* = 6). *Indicates significant differences (*p* < 0.05). Green arrows indicate the positive Z-score implied in an active status, and purple arrows indicate the negative-Z-score.

BioPAN analysis evidenced that the synthesis of PC 16:0_20:5 (PC 36:5) was activated, either through the pathway TG 48:1 → DG 32:1 → PC 32:1 → LPC 16:1 → PC 32:2 → DG 32:2 → TG 52:5 → DG 36:5 → PC 36:5 (Z-score: 3.34) or via the hydrolysis of PE 36:5 → PC 36:5 (Z-score: 3.13) (Figure 4B).

Regarding PEs, the most abundant species contained 38 acyl carbons, representing approximately 53% and 52% of total PEs in 2D and 3D cultures, respectively. Spheroids showed a decrease in shorter-chain PEs (32 and 34 acyl carbons), along with a significant increase in very-long-chain species (42 and 44 acyl carbons; Figure 3C). Among these, the highest increases were observed for PE 44:8 (7.2-fold), PE 42:6 (5.3-fold), and PE 44:4 (4.8-fold). However, changes in the unsaturation levels of PEs were inconclusive, as shown in Figure 3D.

### 3.4 Gene Expression

Gene expression analysis revealed a significant increase in the basal expression levels of genes related to liver function in ZFL spheroids. These included a 2.5-fold increase in *asl,* related to the urea cycle, and a 1.4-fold increase in *g6pd*, associated with liver carbohydrate metabolism (Figure 5A). No changes were observed in *igf1*, *cyp3a65*, or *ugt1a1*, which are involved in growth and development, response to xenobiotic stimuli, and glucuronidation, respectively (Figure 5A).

**Figure 5:**
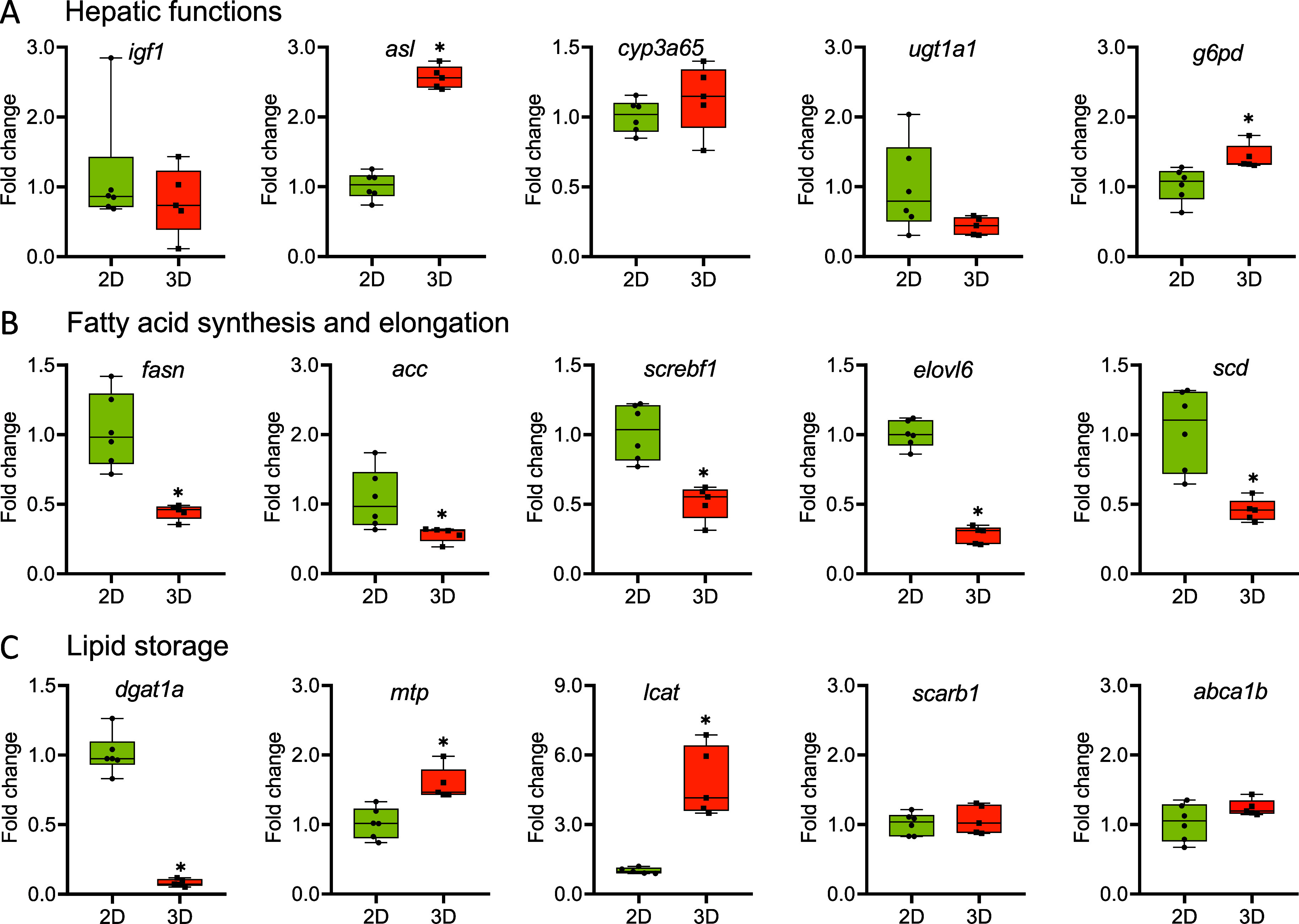
Altered gene expression in ZFL cell monolayers and spheroids associated with hepatic functions, fatty acid synthesis, and lipid storage. *Statistically significant differences (*p* < 0.05).

Several genes involved in fatty acid biosynthesis were downregulated in ZFL spheroids. These include *fasn* (2.2-fold), which plays a key role in the synthesis of the 16-carbon saturated fatty acid palmitate from acetyl-CoA and malonyl-CoA, a central process in lipogenesis; *acc* (1.8-fold), which catalyzes the carboxylation of acetyl-CoA to malonyl-CoA, the rate-limiting step in fatty acid synthesis; *screbf1* (1.9-fold), encoding sterol regulatory element-binding protein 1, essential for liver lipogenesis and the activation of fatty acid synthesis genes; *elovl6* (3.6-fold), which participates in the elongation of saturated and monounsaturated fatty acids with 12, 14, and 16 carbons; and *scd* (2.2-fold), involved in the synthesis of oleic acid (Figure 5B).

Additionally, the expression of *dgat1a* decreased significantly by 12-fold in spheroids. This gene is responsible for converting DGs and fatty acyl-CoA into TGs. In contrast, *mtp* (1.6-fold), which facilitates the transfer of TGs and CEs from the endoplasmic reticulum to lipoprotein particles, and *lcat* (4.8-fold), which encodes the enzyme responsible for extracellular cholesterol esterification, showed increased expression. Meanwhile, the expression of *scarb1* and *abca1b*, which are involved in cholesterol transfer to and from high-density lipoproteins, as well as the regulation of cellular cholesterol and phospholipid homeostasis, remained unchanged (Figure 5C).

Exposure to 1 μM BNF led to a significant increase in *cyp1a* in ZFL spheroids, with a 650-fold increase after 6 h and a 768-fold increase after 24 h, compared to a 57-fold increase in cell monolayers after 6 h (Figure 6).

**Figure 6:**
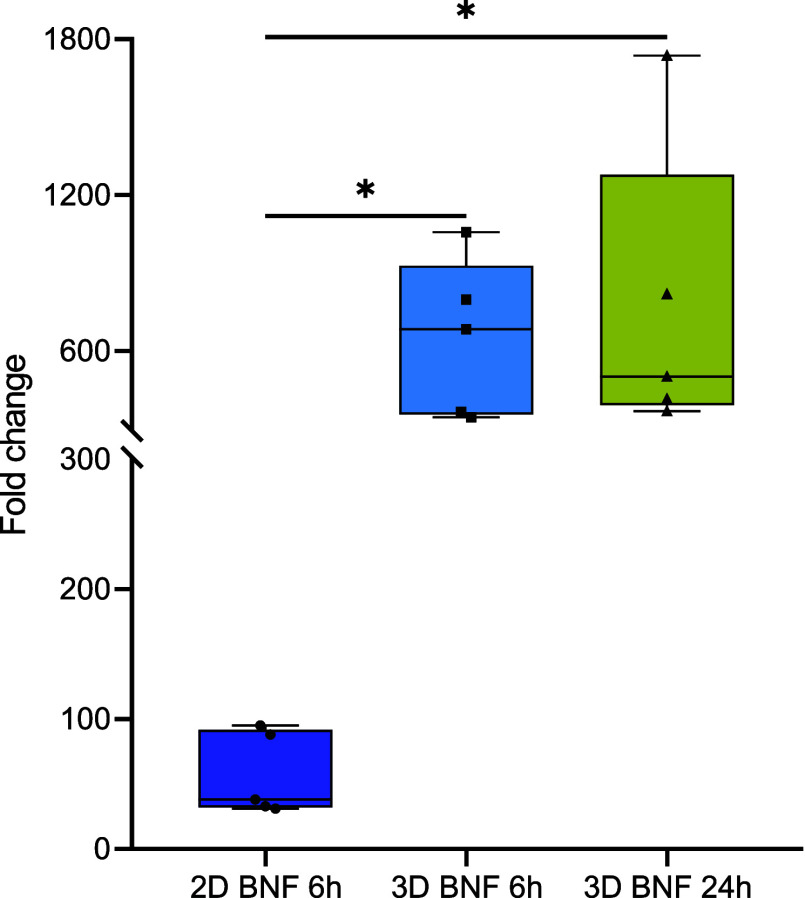
Fold change of *cyp1a* gene expression in ZFL cells after exposure to 1 μM BNF for 6 h (2D and 3D) and 24 h (3D). *Statistically significant differences vs control (*p* < 0.05).

## 4 Discussion

This study provides an in-depth lipidomic characterization of 3D spheroid cultures derived from the ZFL immortalized cell line compared with conventional 2D monolayer cultures. Our findings highlight significant alterations in lipid profiles, fatty acid composition, and the expression of liver-specific genes, particularly those related to lipid metabolism and metabolic competence. The lipidomic profile of ZFL spheroids showed significant differences compared to monolayer cultures: spheroids were enriched in CEs, DGs, LPCs, and Cer, while levels of PEs, ether-linked PEs, ether-linked PCs, and SMs were reduced. These shifts are consistent with previous studies on 3D models (fish liver cells and breast cancer cells), which similarly reported high levels of neutral lipids such as CEs, along with an increased ratio of acylglycerols to membrane lipids, likely associated with the formation of large lipid droplets.^18,31^ Gene expression analysis further supports these observations by showing an upregulation of *lcat*, the gene encoding lecithin-cholesterol acyltransferase, which plays a crucial role in the esterification of cholesterol. This process is essential for the maturation of high-density lipoproteins and the transport of cholesterol from tissues to the liver.^32,33^

Additionally, we observed a downregulation of *dgat1a* expression in ZFL spheroids, suggesting reduced synthesis of TGs from DGs. This is consistent with the observed increase in DG levels in spheroids. In contrast, previous analyses of PLHC-1 spheroids showed slightly lower levels of DGs compared to cell monolayers.^18^ This discrepancy may arise because PLHC-1 cells, being derived from a cancerous origin, may have altered lipid turnover rates, favoring rapid DG utilization for phospholipid or TG synthesis. These metabolic shifts reflect broader changes in lipid handling among cell types, and their direct link to toxicological endpoints requires further investigation.

Importantly, seven-day-old ZFL spheroids, even those exceeding 400 μm in diameter, did not develop a necrotic core (Figure 1). This finding is consistent with previous studies by De Souza et al. (2021),^34^ who reported similar results for the same cell line, as well as with studies on primary human hepatocyte spheroids,^35^ where hypoxic cells were distributed throughout the spheroid structure. In contrast, spheroids of cells from cancerous origins, such as PLHC-1, A549, and MCF-7, often exhibited necrotic or hypoxic cores at sizes greater than 250 μm due to oxygen and nutrient limitations.^18,36,37^ Indeed, this phenomenon appears to be far less common in spheroids derived from noncancerous cells, such as ZFL cells, which is an important feature for their use in toxicological testing.

Although live-dead staining of ZFL spheroids harvested 7 days postseeding did not reveal significant cell death, we observed significant changes in sphingolipid metabolism, with a shift from SMs and SPBs to ceramides (Figure 2). Elevated ceramide levels in liver cells are typically associated with a switch from glucose to lipid metabolism, leading to triglyceride accumulation.^38^ However, no significant increase in triglyceride levels was observed in this study. Ceramides are also known to impair glucose homeostasis by negatively affecting the insulin signaling pathway in organs like the liver.^39^ Interestingly, we found increased expression of *g6pd* in ZFL spheroids compared with cell monolayers. This suggests that the three-dimensional spheroid architecture induces metabolic adaptations, potentially activating pathways like the pentose phosphate pathway to meet the energy and biosynthetic demands of spheroid growth. While these findings imply complex metabolic reprogramming, additional functional studies are required to elucidate their full significance.

Previous research has shown that ZFL spheroids enhance intercellular interactions through E-cadherin, a key component of adherens junctions vital for cell adhesion.^15,40^ Accordingly, we observed significant changes in membrane lipid composition, particularly among PEs and PCs. These include a downregulation of total PEs as well as ether-linked PEs and PCs, accompanied by an upregulation of PUFA-containing PCs and PEs with very long-chain fatty acids, indicating membrane reorganization. These results align with previous studies in PLHC-1 spheroids, which also reported a reduction in membrane lipids associated with enhanced inter- and extracellular interactions, resembling in vivo-like conditions.^18^ Both PLHC-1 and ZFL spheroids exhibited an increased PC/PE ratio compared with their respective 2D cultures, a phenomenon not observed in primary zebrafish hepatocytes grown in 2D (unpublished data). The increased PC/PE ratio hints at specific adaptations linked to 3D organization and potentially reflects a more differentiated, in vivo-like phenotype.

PUFA-containing phospholipids are crucial for maintaining membrane fluidity and facilitating processes like endo/exocytosis.^41–43^ The increased abundance of PUFA-containing PCs, particularly species that are present at very low levels in 2D cultures but markedly elevated in 3D (e.g., PC 16:0_20:5; 16:0_22:5), may contribute to improved membrane properties. Interestingly, Hamano et al. (2021) reported a significant rise in very-long-chain PCs and PEs during the late stages of mouse retinal development. While the role of very-long-chain phospholipids in the liver remains unclear, the increased levels of very-long-chain PEs in 3D cultures may be indicative of greater hepatocyte maturity. These compositional changes at the membrane level likely reflect broader cellular adaptations, including shifts in metabolic activity, which are key indicators of increased functional maturity in 3D cultures.

Previous studies have shown that 3D cultures of cardiomyocytes and hepatocytes exhibit enhanced maturity compared to 2D cultures.^6,45^ Thus, adapting human pluripotent stem cell-derived cardiomyocytes to a 3D environment results in a more mature metabolic profile, characterized by reduced glycolysis and fatty acid synthesis, along with increased tricarboxylic acid (TCA) cycle activity. This suggests a shift in fatty acid metabolism from biosynthesis to oxidation.^45^ Likewise, our findings indicate a decrease in the expression of genes associated with fatty acid synthesis, which reflects changes in fatty acid metabolism and may influence cell growth during the development of a mature phenotype.^8^ Further supporting this, Park et al. (2022)^15^ demonstrated that ZFL spheroids exhibit reduced cell proliferation and replication, along with increased expression of genes linked to glucose and glycogen synthesis and metabolic activity, compared to cell monolayers. Despite these metabolic shifts, the expression of *igf*, a gene encoding a liver-synthesized hormone that regulates cell senescence, survival, and proliferation,^46^ remained similar to that in cell monolayers, indicating that spheroids retain essential homeostatic functions.

Additionally, our results demonstrated a significant increase in the expression of genes involved in the urea cycle, such as *asl*, reinforcing previous observations of enhanced liver-specific functionalities in 3D fish liver cultures. Thus, fish liver spheroids have been shown to exhibit elevated albumin and urea secretion, increased expression of CYP450-related genes, enhanced vitellogenin (Vtg) synthesis, and upregulated expression of ATP-binding cassette (ABC) transporters.^10,15^

One key finding was the remarkably stronger induction of *cyp1a* after 6 h of exposure to 1 μM BNF in spheroids (650-fold) compared with 2D cultures (57-fold). While such robust transcriptional responses are consistent with strong xenobiotic stimulation, we acknowledge that confirming these results at the protein or enzymatic activity level would provide further validation. Unfortunately, technical limitations, including limited biological material due to the use of individual spheroids, prevented these analyses in the current study. However, our findings are supported by previous studies; for example, CYP450 enzymes, such as CYP1A and CYP3A, which are essential for xenobiotic and drug metabolism, have shown activity levels 10 to 1,000 times higher in 3D primary human hepatocytes compared to 2D cultures.^47^ While extensive research has characterized metabolic activity in human 3D hepatocytes, studies on fish liver spheroids (e.g., *cyp1a* induction) remain limited. Jeong et al. (2016) demonstrated that ZFL spheroids cultured via magnetic levitation and exposed to 30 μg/mL carbamazepine showed a 2-fold increase in *cyp1a1* expression compared to 2D cultures, approaching expression levels observed in zebrafish embryos. Similarly, RTL-W1 spheroids (15 days postseeding) showed significantly higher basal and BNF-induced *cyp1a* expression than cell monolayers.^12^ Moreover, Rodd et al. (2017)^11^ found that PLHC-1 spheroids were a sensitive model for liver toxicant assessment, detecting significant *cyp1a* induction and protein expression after 24 h of exposure to 1 nM benzo­(a)­pyrene.

In conclusion, this study provides a comprehensive lipidomic analysis of ZFL spheroids, highlighting key changes in membrane lipid composition and metabolic activity compared with 2D cultures. We observed a reduction in total membrane lipids and an upregulation of polyunsaturated PCs, very-long-chain highly unsaturated PEs, as well as an increase in neutral lipids such as CEs and DGs. These shifts suggest structural adaptations that may influence membrane fluidity, lipid storage, and cell signaling. Gene expression analysis further revealed enhanced cholesterol esterification and alterations in TG metabolism, supporting a metabolic shift in the 3D cultures. Moreover, the downregulation of genes involved in fatty acid biosynthesis, coupled with the significant upregulation of *cyp1a* and urea cycle-related genes, indicates significant changes in energy metabolism and enhanced metabolic competence of ZFL spheroids. Together, these findings make ZFL spheroids a promising model for studying fish liver function, lipid metabolism, and toxicology. Further studies incorporating specific toxicological endpoints are needed to fully validate the predictive capabilities of the model in the assessment of aquatic toxicity.

## Supplementary Material



## Notes

Includes additional experimental details on lipid standards used for quality control and quantification of lipids, as well as the sequences of selected genes; it also includes additional results, such as the list of lipids significantly altered in ZFL spheroids compared to cell monolayers, lipid metabolic reactions activated in spheroids, and statistical analyses of lipidomic differences between the two culture models (PDF)
