# Supplementary material for: Lipidome and Gene Expression Profiling in Zebrafish Liver Spheroids: A 3D Model for Environmental Toxicology Applications
Source: Environ Sci Technol. 2025 Jun 13;59(25):12543–53. doi: 10.1021/acs.est.5c02300 (PMC12224301; doi:10.1021/acs.est.5c02300)
Supplement: Supplementary file 1 [file es5c02300_si_001.pdf]

Supplementary material

Lipidome and Gene Expression Profiling in Zebrafish Liver Spheroids: A 3D Model for Environmental Toxicology Applications

Tiantian Wang[1,2], Miquel Perelló Amorós[1], Cinta Porte[1]*

[1]Environmental Chemistry Department, IDAEA –CSIC-, C/ Jordi Girona, 18-26, 08034 Barcelona, Spain

[2]PhD Program Aquaculture, University of Barcelona. Av. Diagonal 643, 08028 Barcelona, Spain

***Corresponding author:** Cinta Porte; e-mail: cpvqam@cid.csic.es

Summary: 10 pages, 1 figure, 4 tables.

Index

Table S1. Lipid standards used for quality control and quantification of lipid species……..…………………………………………………………………………....3

Table S2. List of genes selected and their sequences………………..…….…………...4

Table S3. List of lipids identified by the volcano plot as significantly changed in spheroids in comparison to cell monolayers……………………....……………........6

Table S4: Lipid reactions activated in ZFL spheroids compared to cell monolayers.....9

Figure S1. Statistical analysis of the lipidome of ZFL cell spheroids *vs.* cell monolayers…………………………………………………………………………10

Table S1. Characteristics of internal lipid standards used for quality control and quantification. The table includes retention time (RT), measured and theoretical *m/z* values, adduct forms, and concentrations (in pmol) of each standard compound.

Name	RT [min]	Meas. *m/z*	Theoretical mass	Adduct ions	Concentration [pmol]
16:0 (d7) CE	17.57	649.6624	631.6286	[M+NH4]+	200
16:0 (d31) Cer	9.49	569.7138	568.7065	[M+H]+	200
15:0-18:1 (d7) DG	11.35	605.5849	587.5509	[M+NH4]+	50
17:0 Lyso PC	4.57	510.3561	509.3487	[M+H]+	50
16:0 (d31)-18:1 PC	10.68	791.7807	790.7727	[M+H]+	200
C18(plasm)-18:1(d9) PC-P	12.55	781.6790	780.6716	[M+H]+	50
16:0 (d31)-18:1 PE	10.67	748.7267	747.7198	[M+H]+	200
16:0 (d31)-18:1 PG	8.8	796.7474	778.7137	[M+NH4]+	200
16:0 (d31)-18:1 PI	8.67	884.7631	866.7295	[M+NH4]+	50
16:0 (d31)-18:1 PS	9.12	792.7147	791.7080	[M+H]+	200
16:0 (d31) SM	9.23	734.7698	733.7626	[M+H]+	100
17:0-17:1-17:0 (d5) TG	17.8	869.8341	851.7995	[M+NH4]+	100

Table S2. Primer sequences and amplification efficiencies for target genes used in qPCR analysis.

Gene name	Primer name	Sequence	Database accession no.	Effciency (%)
Insulin-like growth factor 1	*igf1-F*	GGGGCATTGGTGTGATGTCT	NM_131825.2	91
	igf1-R	ATCCTGTCGGTTTGCTGAAAT		
Argininosuccinate lyase	*asl-F*	TGTTGGATGTGCAGGCTCT	NM_200451.1	77
	asl-R	ACCCCATAGTTTATTTCCCTCTGA		
Cytochrome P450, family 3, subfamily A, polypeptide 65	*cyp3a65-F*	CGGAGAGCTTCAAACCCGA	NM_001037438.1	87
	cyp3a65-R	CCAGACCGAACGGCATGTAC		
UDP glucuronosyltransferase 1 family, polypeptide A1	*ugt1a1-F*	CGTTTGATTCCTTGTCGTCTTG	NM_001037428.2	88
	ugt1a1-R	AACCACTGGAGTAGCGAGGC		
Glucose-6-phosphate dehydrogenase	*g6pd-F*	TTGGACGTCTTTTGTGGCAG	XM_005162010.4	93
	g6pd-R	GTATGTTCCCTCGTAGCGGA		
Fatty acid synthase	*fasn-F*	CGACCGCTACGTTCCCACT	XM_021472581.1	91
	fasn-R	CCATCTCCGTACTCGCTGCT		
Acetyl-CoA carboxylase	*acc-F*	ACCCCAACAGAGGGAACA	XM_017356117	88
	acc-R	AAGAGCGGAAGGAAACCA		
Sterol regulatory element binding transcription factor 1	*srebf1-F*	CCGAAAGCATGTGTGAACGT	NM_001105129.1	90

	srebf1-R	AGTAACTCAGGCTCGGCTGC		
Long chain fatty acid elongase 6	*elovl6-F*	CTATGCTCTTCGGGCAGCC	NM_199532.1	104
	elovl6-R	ACCATCTGGGTGATCTGTGTCA		
Stearoyl-CoA desaturase	*scd-F*	CACCACACGTTTCCCTACGA	NM_198815.2	97
	scd-R	CATGGTGTCCACGAAGATGG		
Diacylglycerol O-acyltransferase 1a	*dgat1a-F*	CTGGGCGTTTCTGGGTATGA	NM_199730.1	94
	dgat1a-R	GCGTTGCCATAGTTACCCCTC		
Microsomal triglyceride transfer protein	*mtp-F*	GAGGCCACGCTGGATTTCAT		87
	mtp-R	TTGGACACCGTCTCTCTGAAG		
Lecithin-cholesterol acyltransferase	*lcat-F*	TCCAAACGCTGACCCCATAG	NM_001324407.1	90
	lcat-R	CCTGCTCCTGCTTCCCAATC		
Scavenger Receptor Class B Member 1	*scarb1-F*	GTCAGCTCCTGCAGACACG	NM_198921.2	77
	scarb1-R	TCTTCACTTGGGCTCAATCC		
ATP-binding cassette, sub-family A member 1b	*abca1b-F*	AGCTCAATCAGCCGTAAAGGG	NM_001114586.2	88
	abca1b-R	GGGCTTCTCCAGGCTGTCTT		
Cytochrome P450, family 1, subfamily A	*cyp1a-F*	GGTTAAAGTTCACCGGGATGC	NM131879.2	93
	cyp1a-R	CTGTGGTGTGACCCGAAGAAG		

Table S3. List of lipids that were significantly changed in ZFL spheroids (7-days post seeding) in comparison with cell monolayers after volcano plot analysis (fold change > 2, p-value < 0.05). Upregulated lipids are marked in orange and downregulated lipids in blue. FC: fold change.

	FC	log2(FC)	raw.pval	-log10(p)
CE 14:0	15.921	3.9929	1.51E-06	5.8203
CE 16:0	10.486	3.3903	3.29E-07	6.4828
CE 16:1	16.733	4.0646	7.24E-07	6.1405
CE 18:0	15.123	3.9187	1.01E-06	5.9961
CE 18:1	11.126	3.4759	9.00E-08	7.0457
CE 18:2	17.457	4.1257	3.07E-07	6.5125
CE 18:3	21.024	4.394	9.71E-07	6.0127
CE 20:1	11.847	3.5664	8.41E-07	6.0754
CE 20:2	6.1683	2.6249	2.30E-06	5.6385
CE 20:3	14.273	3.8353	1.09E-06	5.9637
CE 20:4	21.709	4.4402	1.16E-06	5.9338
Cer 18:0;O2/16:0	8.9196	3.157	5.14E-05	4.289
Cer 18:0;O2/24:0	25.433	4.6686	4.62E-05	4.3357
Cer 18:0;O2/24:1	11.037	3.4643	2.52E-07	6.5986
Cer 18:1;O2/16:0	2.9679	1.5694	0.00011551	3.9374
Cer 18:1;O2/24:0	5.0799	2.3448	6.88E-05	4.1624
Cer 18:1;O2/26:0	5.9319	2.5685	0.00026318	3.5797
Cer 18:1;O2/26:1	3.8526	1.9458	2.97E-06	5.5279
Cer 32:0;O2	3.8685	1.9518	4.31E-05	4.3656
Cer 34:0;O2	8.3095	3.0548	3.06E-06	5.5146
Cer 34:0;O3	2.1032	1.0726	0.0001076	3.9682
Cer 36:0;O2	5.0473	2.3355	0.00062689	3.2028
Cer 40:0;O2	8.0935	3.0168	5.34E-06	5.2724
Cer 40:1;O2	3.3289	1.735	6.05E-07	6.2185
Cer 44:0;O2	9.0174	3.1727	0.0014184	2.8482
Cer 44:1;O2	17.979	4.1682	5.09E-06	5.2937
Cer 44:2;O2	6.0518	2.5974	4.08E-06	5.3889
DG 16:0/16:0	0.4146	-1.2702	1.59E-08	7.7994
DG 30:0	0.32486	-1.6221	5.51E-07	6.2587
DG 30:1	0.40289	-1.3116	0.01815	1.7411
DG 18:0_18:1	2.6875	1.4263	3.56E-08	7.4484
DG 40:8	2.0664	1.0471	0.001938	2.7126
HexCer 18:1;O2/18:0	3.3197	1.731	0.009442	2.0249
HexCer 18:1;O2/24:1	3.2354	1.6939	1.31E-05	4.8817
HexCer 40:1;O2	5.5078	2.4615	4.15E-05	4.382
HexCer 44:2;O2	19.221	4.2646	1.40E-05	4.8529
LPC 16:0	3.8267	1.9361	9.21E-07	6.0357
LPC 20:3	2.3761	1.2486	0.00014642	3.8344

PC 14:0/14:0	0.31452	-1.6688	0.00012028	3.9198
PC 14:0_16:0	0.23979	-2.0601	5.43E-05	4.2654
PC 14:0_16:1	0.33166	-1.5922	0.00012427	3.9056
PC 16:0_16:2	0.47469	-1.0749	0.00011215	3.9502
PC 16:1_18:2	0.48476	-1.0447	0.00037791	3.4226
PC 30:2	0.17238	-2.5363	5.09E-06	5.2935
PC 32:4	0.32429	-1.6246	0.00026673	3.5739
PC 14:0_22:6	2.0173	1.0124	4.57E-05	4.3404
PC 16:0_20:4	2.7433	1.4559	9.35E-05	4.0292
PC 16:0_20:5	14.326	3.8405	4.36E-07	6.3605
PC 16:0_22:5	10.577	3.4029	5.76E-06	5.2396
PC 18:0_20:4	4.6385	2.2137	4.29E-05	4.3673
PC 18:0_22:6	3.2958	1.7206	0.00010087	3.9962
PC 18:2_20:4	10.944	3.452	8.00E-07	6.0969
PC 20:4/20:4	2.1773	1.1226	9.94E-06	5.0028
PC O-30:0	0.36088	-1.4704	4.63E-10	9.3344
PC O-30:1	0.3964	-1.335	2.30E-07	6.6387
PC O-32:0	0.30765	-1.7006	6.45E-11	10.19
PC O-32:2	0.37507	-1.4148	1.56E-08	7.8065
PC O-34:0	0.44627	-1.164	0.00026928	3.5698
PC O-34:1	0.46381	-1.1084	2.66E-05	4.5749
PC O-34:3	0.43239	-1.2096	3.19E-07	6.4966
PC O-34:5	0.3936	-1.3452	5.64E-05	4.2484
PC O-38:1	0.39005	-1.3583	2.73E-05	4.5635
PC O-38:2	0.32465	-1.6231	1.02E-05	4.9918
PC O-38:3	0.25312	-1.9821	9.33E-07	6.03
PC O-38:4	0.34847	-1.5209	1.04E-08	7.9844
PC O-40:1	0.24985	-2.0009	4.74E-05	4.3242
PC O-40:2	0.38188	-1.3888	3.28E-06	5.4842
PC O-40:3	0.37859	-1.4013	4.23E-06	5.3733
PC O-40:4	0.29495	-1.7615	1.59E-07	6.7989
PC O-42:7	0.22508	-2.1515	1.02E-09	8.9924
PE 32:0	0.40862	-1.2912	0.00020429	3.6898
PE 32:1	0.42507	-1.2342	0.00015868	3.7995
PE 32:2	0.47131	-1.0853	0.0056231	2.25
PE 34:0	0.45288	-1.1428	2.75E-06	5.5602
PE 34:3	0.34236	-1.5464	3.04E-05	4.5169
PE 36:3	0.37105	-1.4303	2.59E-05	4.5866
PE 36:4	0.37969	-1.3971	4.32E-06	5.3646
PE 36:5	0.33915	-1.56	1.52E-06	5.8179
PE 38:1	0.39353	-1.3455	6.47E-06	5.1889
PE 38:5	0.30679	-1.7047	4.11E-07	6.3861
PE 38:6	0.44934	-1.1541	1.03E-05	4.9879
PE 38:7	0.30045	-1.7348	1.81E-07	6.7433
PE 40:6	0.45944	-1.1221	6.39E-07	6.1944
PE 40:8	0.4284	-1.223	1.90E-08	7.721

PE 42:4	2.6158	1.3873	0.00093006	3.0315
PE 42:5	2.1076	1.0756	0.034309	1.4646
PE 42:6	5.3431	2.4177	0.0027392	2.5624
PE 42:8	2.8943	1.5332	0.000908	3.0419
PE 44:4	4.8519	2.2785	0.032273	1.4912
PE 44:6	4.3365	2.1165	0.0027985	2.5531
PE 44:8	7.2379	2.8556	0.041871	1.3781
PE O-32:0	0.4642	-1.1072	0.00029775	3.5261
PE O-34:3	0.40577	-1.3013	0.00058594	3.2321
PE O-34:4	0.37257	-1.4244	7.02E-05	4.1535
PE O-36:3	0.43355	-1.2057	0.0015523	2.809
PE O-36:4	0.27123	-1.8824	6.11E-07	6.2138
PE O-36:5	0.42191	-1.245	9.51E-05	4.022
PE O-38:2	0.48104	-1.0558	4.03E-06	5.3943
PE O-38:3	0.16383	-2.6097	4.26E-05	4.3704
PE O-38:4	0.39322	-1.3466	0.00079032	3.1022
PE O-38:5	0.21929	-2.1891	4.11E-06	5.3862
PE O-38:6	0.29277	-1.7722	9.78E-08	7.0095
PE O-40:4	0.40091	-1.3187	0.0017055	2.7682
PE O-40:5	0.29342	-1.769	1.71E-05	4.7677
PE O-40:8	0.46239	-1.1128	5.24E-05	4.2803
PE O-42:7	0.3858	-1.3741	7.64E-05	4.117
PG 36:3	0.22785	-2.1339	0.021268	1.6723
PG 38:3	0.46197	-1.1141	0.0022352	2.6507
PI 34:1	3.1087	1.6363	0.00027871	3.5549
PS 30:1	3.5773	1.8389	6.13E-06	5.2122
PS 32:3	0.26953	-1.8915	0.0023182	2.6348
SM 18:1;O2/22:1	0.168	-2.5734	1.08E-08	7.9686
SM 18:1;O2/24:1	0.4509	-1.1491	4.94E-06	5.3059
SM 18:1;O2/24:2	0.28504	-1.8108	2.58E-07	6.5883
SM 34:1;O3	0.48866	-1.0331	0.0001976	3.7042
SM 38:1;O2	0.42725	-1.2268	9.61E-06	5.0175
SM 18:1;O2/24:0	2.1593	1.1106	0.00089263	3.0493
SM 42:0;O2	8.8517	3.146	5.43E-06	5.265
SM 44:1;O2	7.6385	2.9333	1.28E-05	4.8931
TG 46:3	0.31532	-1.6651	2.29E-06	5.6399
TG 48:10	0.35188	-1.5069	4.94E-08	7.3063
TG 48:2	0.44248	-1.1763	4.52E-06	5.3447
TG 48:3	0.26464	-1.9179	3.79E-07	6.4217
TG 48:4	0.45322	-1.1417	0.00063008	3.2006
TG 50:4	0.48896	-1.0322	0.00010545	3.977
TG 60:8	0.30896	-1.6945	3.94E-05	4.404
TG 50:6	0.37084	-1.4311	0.01631	1.7875
TG 16:0_18:0_20:1	2.4987	1.3212	0.012381	1.9072
TG 60:10	2.4955	1.3194	0.001572	2.8035
TG 60:11	5.2751	2.3992	2.11E-06	5.6754

Table S4. BioPan analysis evidenced the lipid reactions that were activated in ZFL spheroids compared to cell monolayers.

**Reactions chains**	**Z-score**	**Predicted genes**
PE→PC→LPC	4.275	*PEMT*, *PLA2G2E*, *PLA2G2A*, *PLA2G2D*, *PLA2G2F*, *PLA2G1B*, *PLA2G4A*, *PLA2G4B*, *PLA2G4C*, *PLA2G4D*, *PLA2G4E*, *PLA2G4F*
PE→PC→DG	3.999	*PEMT*
SPB→Cer	3.791	*CERS1*, *CERS2*, *CERS3*, *CERS4*, *CERS5*, *CERS6*, *ASAH1*, *ASAH2*, *ASAH2B*
PE→PC→PS	3.637	*PEMT*, *PTDSS1*
dhSPB→dhCer	3.15	*CERS1*, *CERS2*, *CERS3*, *CERS4*, *CERS5*, *CERS6*
PC→LPC	3.073	*PLA2G2E*, *PLA2G2A*, *PLA2G2D*, *PLA2G2F*, *PLA2G1B*, *PLA2G4A*, *PLA2G4B*, *PLA2G4C*, *PLA2G4D*, *PLA2G4E*, *PLA2G4F*
PC→DG	2.683	No genes have yet been identified
SM→Cer	2.594	*SMPD1*, *SMPD4*
TG→DG	2.366	*PNPLA4*, *PNPLA5*
dhSM→dhCer	2.343	*SGMS1*, *SGMS2*, *SMPD1*
PC→PS	2.171	*PTDSS1*
PE→PS	2.137	*PTDSS2*

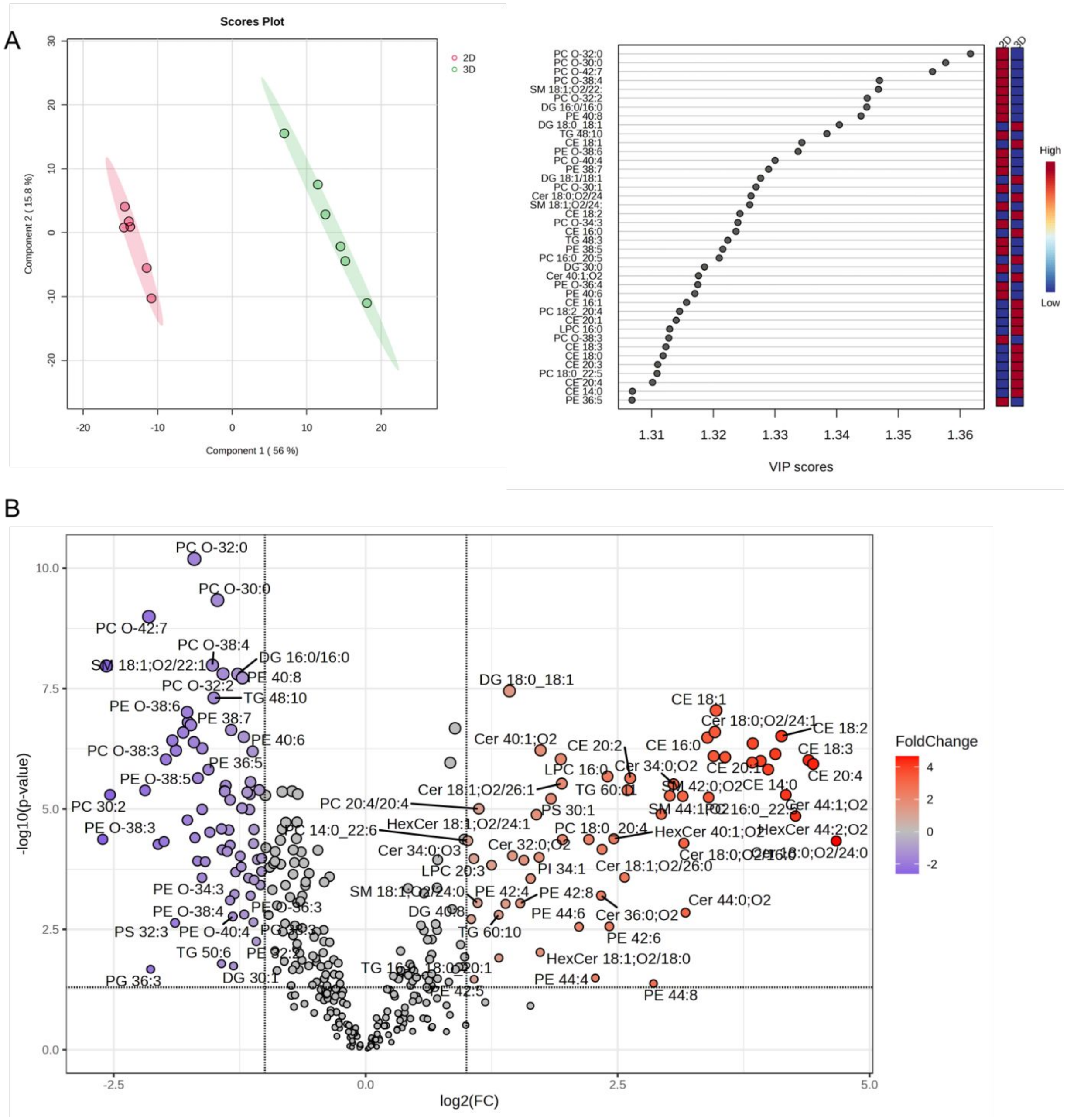

Figure S1. Analysis of the lipidome of ZFL cell spheroids vs cell monolayers. (A) PLS-DA scores plot shows clear discrimination among the lipidome of 3D spheroids and cell monolayers (2D). (B) The volcano plot highlights a large number of individual lipids significantly up- or down-regulated in ZFL spheroids when compared with cell monolayers (FC > 2; $p < 0.05$). Red dots: up-regulated lipids; blue dots: down-regulated lipids; gray dots: no significant differences.
